# Visible to near-infrared supercontinuum generation in yttrium orthosilicate bulk crystal and ion implanted planar waveguide

**DOI:** 10.1038/srep31612

**Published:** 2016-08-16

**Authors:** Bingxi Xiang, Xikui Ren, Shuangchen Ruan, Lei Wang, Peiguang Yan, Huangpu Han, Meng Wang, Jinde Yin

**Affiliations:** 1 Shenzhen Key Laboratory of Laser Engineering, College of Optoelectronic Engineering, Shenzhen University, Shenzhen 518060, China; 2 Key Laboratory of Optoelectronic Devices and Systems of Ministry of Education and Guangdong Province, College of Optoelectronic Engineering, Shenzhen University, Shenzhen 518060, China; 3 School of Physics, Shandong University, Jinan 250100, China

## Abstract

This paper reports on the supercontinuum generation in yttrium orthosilicate bulk crystal and 6-mm-long ion implanted planar waveguide. The waveguide is fabricated by 6 MeV oxygen ions implantation with fluence of 5 × 10^14^ ions/cm^2^ at room temperature. The yttrium orthosilicate bulk crystal and waveguide are pumped using a mode-locked Ti:Sapphire laser with a center wavelength of 800 nm. The generated broadest supercontinuum spans 720 nm (at −30 dB points) from 380 to 1100 nm in bulk crystal and 510 nm (at −30 dB points) from 490 to 1000 nm in ion implanted waveguide, respectively. Compared to the bulk crystal, the ion implanted waveguide requires almost three orders of magnitude lower pump power to achieve a similar level of broadening. The supercontinuum is generated in the normal dispersion regime and exhibits a relatively smooth spectral shape. Our research findings indicate that ion implantation is an efficient method to produce waveguide in yttrium orthosilicate crystal for low-threshold supercontinuum generation.

Supercontinuum (SC) generation is a large spectral broadening in condensed media by intense laser pulses. It has attracted tremendous attentions in recent years^1^,^2^,^3^,^4^ due to its wide applications to optical frequency combs generation^5^,^6^,^7^,^8^, optical coherence tomography^9^,^10^, spectroscopy^11^ and tunable multi-wavelength laser sources^12^. Additionally, the SC covering the visible to near-infrared spectral window possesses low absorption and scattering coefficients in tissue and cells, therefore can offer distinctive advantages for bio-imaging^13^ and Raman spectroscopy^14^.

More recently, broadband integrated SC sources have been reported^15^, which can potentially lead to integrated devices with compact footprints for effective cost reduction in mass-production. These integrated SC sources are constructed based on the optical waveguide technology. Waveguide structures can confine light propagation to small dimensions of few microns, and the increased confinement can enhance the nonlinearity of the waveguide. Waveguide-based SC, covering broad spectral region ranging from visible to mid-infrared, has been realized in a number of materials, such as silicon^16^,^17^,^18^,^19^, silicon nitride^15^,^20^,^21^,^22^, silica^23^, chalcogenide^24^,^25^,^26^,^27^, high-index-doped silica^28^, amorphous silicon^29^,^30^, silicon germanium^31^ and periodically poled lithium niobate^32^.

A basic challenge in the realization of SC in waveguides is the fabrication of high-quality waveguide structures. A few techniques have been developed to produce waveguides in optical materials for SC generation, including film deposition^22^, ion exchange^32^ and ultrafast laser inscription^27^, etc. As an alternative method of fabricating waveguide structures, ion implantation is a mature and commonly-used technology, possessing several advantages for waveguide structure fabrication. The main advantages of ion implantation are: (1) Wide applicability of materials, ion implantation has been successfully used to form waveguide structures in more than 100 materials, including single crystals, glasses, polycrystalline ceramics, and organic materials^33^,^34^; (2) Precise control of the refractive index profile (RIP), the index profile of waveguide can be precisely controlled in selected regions with large refractive indices modulations; (3) Waveguide can be fabricated at low temperature, which ensures steady chemical compositions and phases in the waveguide region^35^; (4) Facilitate its combination with other techniques, waveguide can be fabricated by combining the ion implantation with other techniques, such as ion exchange and metal ion diffusion^36^,^37^. However, up to date, there is no report on the SC generation in ion implanted waveguide structures.

Yttrium orthosilicate (YSO) crystal with wide transparent range of wavelength, large band-gap (6.14 eV)^38^, high chemical and photochemical stability provides prospective applications in the field of SC generation covering broad spectral region ranging from ultraviolet to mid-infrared. Like some other crystals, YSO thin films can be fabricated by film deposition techniques, such as reactive ion-beam sputter deposition^39^ and sol-gel method^40^. However, ion implantation is still considered to be the most efficient method for YSO waveguide formation. Waveguides with low loss in YSO crystal have been fabricated by O and He ions implantation^41^,^42^,^43^. In this report, we demonstrate SC generation in YSO bulk crystal and O ions implanted YSO planar waveguide, respectively. The SC generation extends from the ultraviolet light wavelength range (380 nm) to the near-infrared spectral range (1100 nm) in bulk and from the cyan wavelength range (490 nm) to the near-infrared spectral range (1000 nm) in the ion implanted waveguide. The SC is smooth and generated in the normal dispersion regime. This paper opens up a new avenue for ion implanted waveguides as compact, low-threshold SC sources for diverse applications.

## Results

### SC generation in YSO bulk crystal

**Figure 1:**
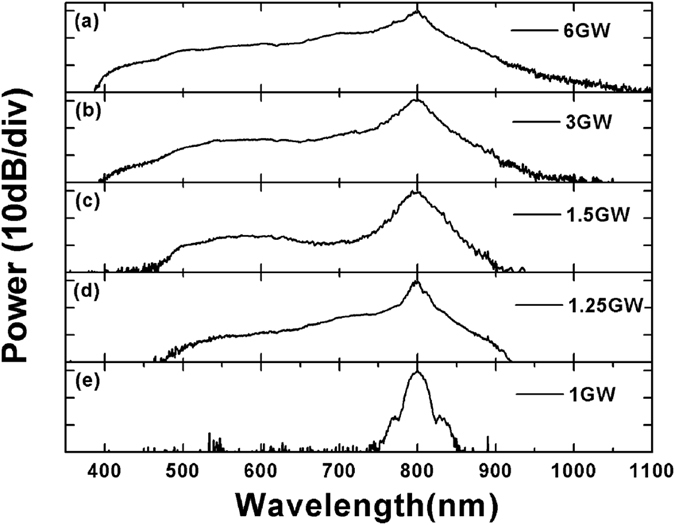
Spectral evolution of the YSO bulk crystal as a function of input beam peak powers.

The experiments of SC generation in YSO bulk crystal are performed using a 100 fs mode-locked Ti:Sapphire laser (Legend Elite, Coherent) with a center wavelength of 800 nm. The collimated laser beam with 500 μm FWHM spot size is launched into a 6-mm-thick YSO crystal. Figure 1 shows the SC spectra of YSO bulk crystal, and the peak powers of the launched pulse are listed in it. At peak power up to 1 GW, the output spectrum is very similar to the input spectrum. At 1.25 GW, the spectrum changes dramatically from a peak still resembling the input spectrum to a very broad, smooth spectrum. With the pulse energy increasing further, broader spectra are formed. At the −30 dB level, the SC at the highest peak power (6 GW) spans 720 nm from 380 to 1100 nm, which is over 1.5 octave of bandwidth.

### SC generation in ion implanted waveguide

**Figure 2:**
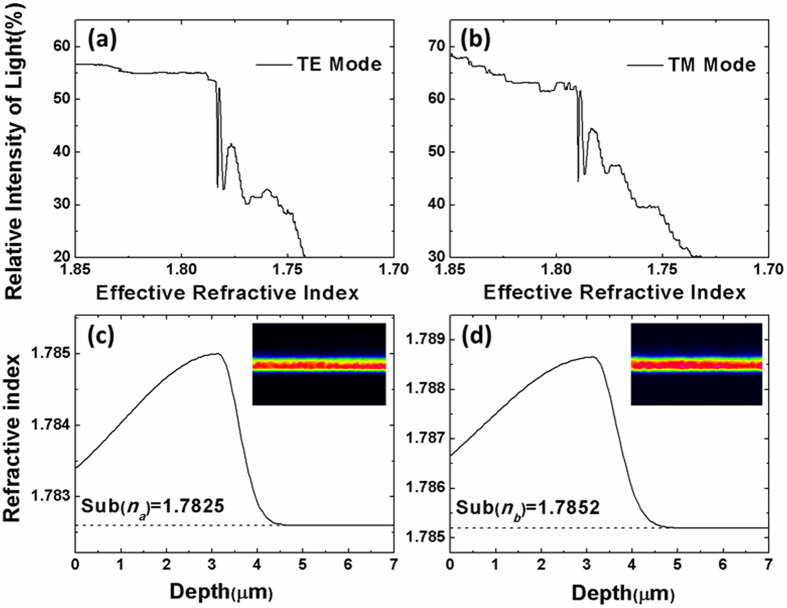
(**a,b**) Dark mode spectra of TE and TM modes in the YSO planar waveguide. (**c,d**) The reconstructed refractive index profile n_a_ and n_b_ of the planar waveguide. The insets in (**c,d**) show the near-field intensity of TE and TM modes.

The YSO planar waveguide is fabricated by O ions implantation. After the ion implantation, thermal annealing treatment is performed to remove the color centers and improve the waveguide quality. Figure 2 (a,b) show dark mode spectra of the TE and TM modes in the YSO waveguide measured at wavelength of 632.8 nm. When the light is coupled into the waveguide region, a lack of the reflected light will result in a dip that may correspond to a propagation mode. As shown in Fig. 2(a,b), the effective refractive indices (n_*eff*_) of the two fundamental modes (TE_0_ and TM_0_) are both higher than those of the substrates. The other dips, which are broader and shallower than the fundamental mode, cannot guide lights. The refractive index profile of the YSO planar waveguide of n_a_ and n_b_ at 632.8 nm is shown in Fig. 2(c,d). Here, two half-Gaussian curves are used to reconstruct the refractive index profile, which is reported by our previous work^41^. The refractive index profile shows a slight increase at the surface that starts to increase to a maximum at a depth of 3.2 μm.

The insets in Fig. 2(c,d) demonstrate the near-field intensity distribution of TE and TM modes at the wavelength of 632.8 nm. It is found that the fundamental TE and TM modes are sufficiently confined in the waveguide. The transmission loss of the waveguide is measured to be around 0.35 dB/cm along the TE mode direction and 0.4 dB/cm along the TM mode direction at wavelength of 632.8 nm using the back-reflection method^44^.

As is widely known, dispersion is the key factor in SC generation. It is difficult to measure the dispersion in planar waveguide. The dispersion is calculated using the finite difference beam propagation method (FD-BPM) in our work. Taking into account the material dispersion, the refractive index profile *N(λ, z)* of the planar waveguide at different wavelengths λ can be estimated by the following equation^45^:


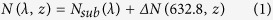


Using the FD-BPM, the effective refractive indices (n_*eff*_) of the TE_0_ and TM_0_ modes at different wavelengths *λ* can be calculated.

**Figure 3:**
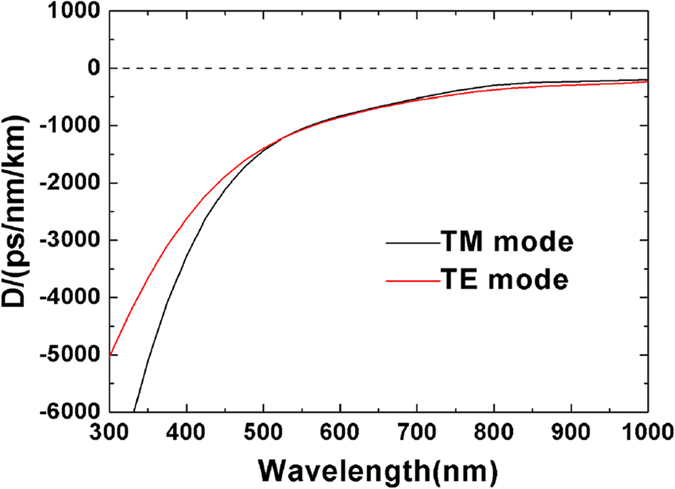
Dispersion curves of TE and TM modes in the YSO planar waveguide.

The dispersion curves of TE_0_ and TM_0_ modes in the YSO planar waveguide are depicted in Fig. 3. For the fundamental TE and TM modes, the dispersion is normal over the entire concerned wavelengths.

**Figure 4:**
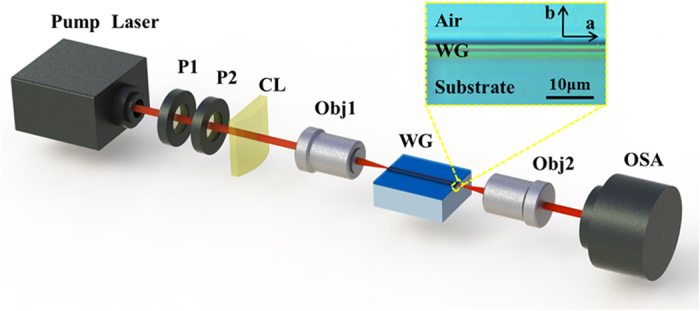
Experimental setup of SC generation in planar waveguide. P1: half-wave plate; P2: polarizer; CL: cylindrical lens; Obj1: objective; WG: YSO planar waveguide; Obj2: objective; OSA: optical spectrum analyzer. The inset shows a microscope photograph (1000×) of the cross-section of a planar waveguide.

Figure 4 shows the experimental setup for SC generation in ion implanted waveguide. A mode-locked Ti:Sapphire laser (Legend Elite, Coherent) is used as a pump source. The pulses are coupled into the YSO waveguide using a combination of cylindrical lens and a 20× microscope objective (NA = 0.4). In this way an elliptical spot is generated on the input facet of the waveguide, thus a well collimated beam is generated propagating inside the planar waveguide with negligible diffraction along the transverse direction. The inset in Fig. 4 shows a photograph, which is collected by a metallographic microscope with 1000× magnification using reflected polarized light of the O ions implanted YSO crystal. The modified layer is approximately 4 μm in the thickness.

**Figure 5:**
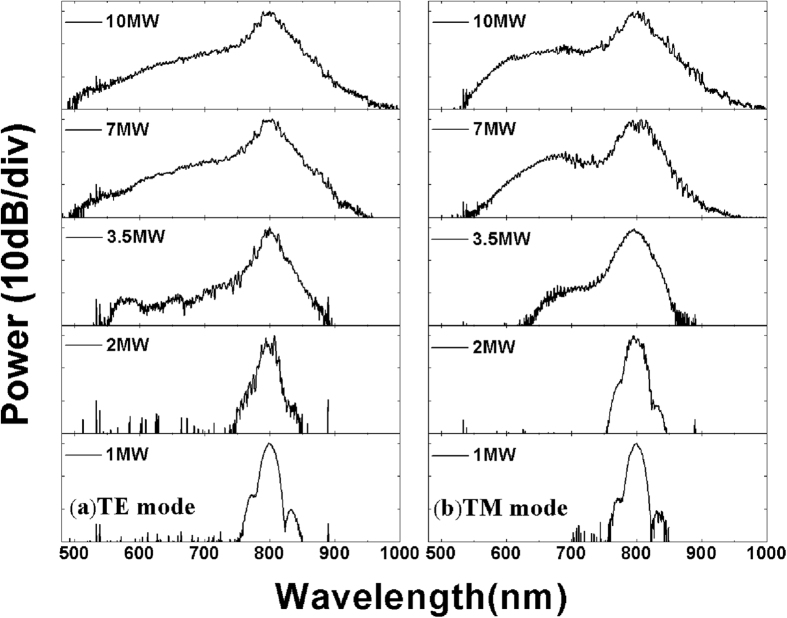
Evolution of the spectra as a function of the peak powers coupled into the waveguide of TE and TM modes.

Figure 5 shows the spectra of 800 nm laser pulses after passing through 6-mm-long ion implanted YSO planar waveguide of TE and TM modes, as a function of the peak powers coupled into the waveguide. As shown in Fig. 5(a), at a low peak power of 1 MW, the spectral width is the same as that of the input pulse. At 2 MW, small ripples emerge in the center of the pulse, which is attributed to self-phase modulation (SPM). At 3.5 MW, the spectrum changes dramatically from a peak similar to the input spectrum to a very broad spectrum. At the maximal available coupled power of 10 MW, the −3 dB bandwidth spans 40 nm from 783 to 823 nm and the −30 dB bandwidth spans 510 nm from 490 to 1000 nm, more than an octave. As we can see, the waveguide generates a relatively narrower bandwidth, compared to the bulk crystal. Further increase the peak power will improve the bandwidth in waveguide, but it is limited by the damage occurring at the input facet. A similar result is also obtained for the TM mode, at the maximal available coupled power of 10 MW, the −30 dB bandwidth spans 470 nm from 530 to 1000 nm, slightly less than the bandwidth of the TE mode.

## Discussion

We have demonstrated a visible to near-infrared SC generation in both YSO bulk crystal and O ions implanted waveguide. The generated SC spectra spanning 720 nm (from 380 to 1100 nm) in YSO bulk crystal and 510 nm (from 490 to 1000 nm) in waveguide have been observed, with relatively smooth spectra. As shown in Fig. 3, the dispersion of TE_0_ and TM_0_ modes is normal over the entire concerned wavelength. As a result, the broadening in waveguide is mainly determined by SPM and cross phase modulation (XPM). It is interesting that strongly asymmetric broadening is observed in the SC spectra. The blue-edge of the spectra broadens much more than the red-edge, indicating that the observed SC spectra cannot be simply explained by SPM and XPM theories, the contribution from the enhancement of SPM by free electrons generated through multiphoton excitation (MPE) should also be specified^46^,^47^,^48^,^49^. The appearance of free electrons (at the peak of the intensity spike) induces a dramatic decrease in the refraction index, thus, leading to a sudden drop in nonlinear phase, which causes a large anti-Stokes broadening by SPM^46^.

Specifically, ion implantation presents some advantages for SC generation compared to another widely used waveguide fabrication technique, ultrafast laser inscription. For instance, ridge waveguide, an important basic structure of high density integrated optics, can be formed by using ion implantation and selective etching^50^,^51^, which offers small size in cross-sectional dimensions, high optical density and small bending radii of curved waveguide due to its large index contrast to the surrounding air. Furthermore, the refractive index profiles of the waveguides can be easily controlled by adjusting the species, energies, fluences and geometries of the implanted ions^33^. This can be potentially used to control the waveguide dispersion with careful waveguide design.

Our work paves a solid way for broader-scope applications for SC generation. On one hand, combined with photolithography and etching technology, highly compressed devices for SC generation can be realized to satisfy some special requirements for particular applications, such as Y-Branch and micro-ring. On the other hand, ion implanted waveguide can be applied to other materials to obtain SC. For example, if the platforms of chalcogenide glass or tellurite glass are used, the generated SC wavelength can be extended to mid-infrared. These new miniature SC sources can be utilized in a wide range of applications for both scientific research and human life.

To the best of our knowledge, this work constitutes the first SC generation in ion implanted waveguides. Results reported in this paper demonstrate that ion implantation is an efficient method to fabricate waveguide in YSO crystal for broad SC generation.

## Methods

### Fabrication of the waveguide

The optically polished YSO crystal used in this work is cut into wafers with dimensions of 10 × 1 × 10 mm^3^ (*a* × *b* × *c* axis). 6 MeV O ions with ion fluence of 5 × 10^14^ ions/cm^2^ are implanted into the optically polished facet (10 × 10 mm^2^) of the crystal plates. The implantations are performed with a 1.7 MV tandem accelerator in Peking University. The ion beam is scanned electrically to ensure a uniform implantation. To prevent channeling effects, the sample is tilted 7° off the direction of the incident O ions beam. After the implantation, the samples are annealed in atmosphere at 200 °C for 30 min.

### Waveguide properties measurement

The dark modes of the formed waveguide are measured by prism coupling. A Metricon prism coupler (Model 2010) fitted with rutile prism is used in the prism-coupling measurements at wavelength of 632.8 nm. During the measurement, a rotary table held the prism and sample could adjust the incident angle of light to reach the coupling conditions, resulting in a dip (mode lines) in the intensity spectrum of the reflected light. An end-face coupling setup is utilized to investigate the near-field mode profiles. The end faces of the waveguide are optically polished so that the light can be coupled into the waveguide by the objective lens. Another objective lens is used to image the near-field mode intensity of the waveguide onto a CCD camera.

### Refractive index distribution of YSO waveguide

Two half-Gaussian curves are used to reconstruct the refractive index profile based on the lattice damage profile that is simulated using the SRIM2013 program (The Stopping and Range of Ions in Matter)^52^. Through varying the heights and widths of the two half-Gaussian curves, the refractive index profile at 632.8 nm wavelength can be obtained when the calculated indices of the modes matched with the indices of the measured modes.

### SC generation in YSO bulk crystal

A mode-locked Ti:Sapphire laser (Legend Elite, Coherent) is used with a center wavelength of 800 nm. The laser provides pulses with about 100 fs duration, a repetition rate of 1 kHz, and a maximum average output power of 4 W. The collimated laser beam with about 500 μm FWHM spot size is launched into the 6-mm-thick YSO crystal at normal incidence along the direction of thickness. The SC spectrum is recorded using an Avantes AvaSpec-3648 fiber optic spectrometer.

### SC generation in waveguide

The mode-locked Ti:Sapphire laser (Legend Elite, Coherent) is used as a pump source. A half-wave plate (P1) and a polarizer (P2) adjust the input power and polarization. The pulses are coupled into the YSO waveguide with a coupling loss of about 3 dB using a combination of cylindrical lens (with about focal distance to the objective lens) and a 20 ×  objective (NA = 0.4). In this way an elliptical spot (with dimensions about 4 um × 100 um, FWHM) is generated on the input facet of the waveguide. The SC is collected using a 40× objective (NA = 0.65) and recorded using an optical spectrum analyzer (AvaSpec-3648). The schematic plot of the experimental setup is depicted in Fig. 4.

## Additional Information

**How to cite this article**: Xiang, B. *et al*. Visible to near-infrared supercontinuum generation in yttrium orthosilicate bulk crystal and ion implanted planar waveguide. *Sci. Rep.* **6**, 31612; doi: 10.1038/srep31612 (2016).
